# A risk score based on pediatric sequential organ failure assessment predicts 90-day mortality in children with *Klebsiella pneumoniae* bloodstream infection

**DOI:** 10.1186/s12879-020-05644-w

**Published:** 2020-12-02

**Authors:** Shuang Li, Jingxian Liu, Feng Chen, Kang Cai, Jintong Tan, Wei Xie, Rong Qian, Xiaoqin Liu, Wenhong Zhang, Huimin Du, Ying Liu, Lisu Huang

**Affiliations:** 1 Department of Infectious Diseases, Xinhua Hospital, Shanghai Jiao Tong University School of Medicine, No. 1665, Kongjiang Road, Yangpu District, Shanghai, 200092 China; 2 Division of Medical Microbiology, Department of Clinical Laboratory, Xinhua Hospital, Shanghai Jiao Tong University School of Medicine, No. 1665, Kongjiang Road, Yangpu District, Shanghai, 200092 China; 3 Department of Neonatal Medicine, Xinhua Hospital, Shanghai Jiao Tong University School of Medicine, No. 1665, Kongjiang Road, Yangpu District, Shanghai, 200092 China; 4 Department of Pediatrics Intensive Care Unit, Xinhua Hospital, Shanghai Jiao Tong University School of Medicine, No. 1665, Kongjiang Road, Yangpu District, Shanghai, 200092 China; 5 Department of Hospital Infection Management, Xinhua Hospital, Shanghai Jiao Tong University School of Medicine, No. 1665, Kongjiang Road, Yangpu District, Shanghai, 200092 China; 6 The National Center for Register-based Research, Aarhus University, Fuglesangs Allé 26, 8210 Aarhus, Denmark; 7 Department of Infectious Diseases, Huashan Hospital, Fudan University, No. 12. Middle Urumqi Road, Jingan District, Shanghai, 200040 China

**Keywords:** *Klebsiella pneumoniae*, Children, Mortality, Risk score

## Abstract

**Background:**

*Klebsiella pneumoniae* bloodstream infection (Kp-BSI) is a serious threat to pediatric patients. The objective of this study was to explore the risk factors, validate the prediction efficiency of pediatric Sequential Organ Failure Assessment (SOFA) and establish better early predictors of mortality in pediatric patients with Kp-BSI.

**Methods:**

All children diagnosed with Kp-BSI were included in this retrospective cohort study from January 2009 to June 2019. Basic characteristics, symptoms and physical examinations, treatments, laboratory statistics, and SOFA at the onset of Kp-BSI were recorded. The Cox proportional hazard model and receiver operating characteristic curves were used to assess the association between the variables and the 90-day mortality and their predictive value. DeLong’s test of receiver operating characteristic curves and integrated discrimination improvement index were used to determine the improvement in predictive capacity of the modified SOFA models. A predictive score was developed using multivariate logistic regression.

**Results:**

Of the 146 children enrolled, 33 (22.6%) patients died within 90 days. Hospitalization in the last 6 months, intra-abdominal source of infection, presence of organ failure, and altered levels of blood biomarkers, including C-reactive protein, albumin, and lactate were significant risk factors for 90-day mortality. The area under the curve (AUC) of SOFA for predicting 90-day mortality was 0.80 (95% CI 0.71–0.89). Moreover, we found that a prediction model combining SOFA with two other parameters, namely hospitalization in the last 6 months and intra-abdominal source of infection, was better at predicting mortality (AUC = 0.89, 95% CI 0.82–0.96; sensitivity = 0.86; specificity = 0.84). According to this novel risk model, we defined three statistically different groups: low-risk, medium-risk and high-risk groups, with an observed 90-day mortality of 5.4, 35.7, and 72.0%, respectively. With reference to the low-risk patients, the medium-risk and high-risk groups had a higher mortality, with hazard ratios of 8.36 (95% CI 3.60–27.83) and 20.27 (95% CI 7.47–54.95), respectively.

**Conclusions:**

The modified SOFA may be better than the original score to predict 90-day mortality in pediatric patients with Kp-BSI. Future prospective studies are required to validate this novel scoring system in external cohorts.

**Supplementary Information:**

The online version contains supplementary material available at 10.1186/s12879-020-05644-w.

## Background

Infections caused by *Enterobacteriaceae*, especially the carbapenem-resistant *Enterobacteriaceae*, which can cost up to $66,031 per patient for hospital treatments in the US [1], are currently considered an urgent threat to global public health. *Klebsiella pneumoniae* (*K. pneumoniae*), a member of the family *Enterobacteriaceae*, is an opportunistic pathogen mainly associated with nosocomial infections [2]. In addition to the increased healthcare expenditure, *K. pneumoniae* is associated with high mortality and prolonged hospital stays [3]. Compared with the overall mortality owing to pneumonia (0.65%) [4], the mortality caused by *K. pneumoniae* bloodstream infection (Kp-BSI) in young children is even higher, ranging from 12.3 to 26.6% in individual institutions [5, 6]. Notably, bloodstream infections by *K. pneumoniae* are more deadly than any other type of infection caused by this species of bacteria [7]. For this reason, instruments with high sensitivity and specificity for early prediction of survival may aid in the timely initiation of treatments and improve the outcome of patients with Kp-BSI.

There have been several studies focused on the early prediction of mortality in children with Kp-BSI. It was reported that intensive care admission, severity of comorbidities, and carbapenem resistance may be independent predictors [5, 6, 8]. The mortality rate in patients with bloodstream infections due to carbapenem-resistant *K. pneumoniae* (CR-Kp) was found to be eight times higher than in those infected with carbapenem-susceptible strains [6]; in other studies, however, the results were inconsistent [5, 9]. Additionally, the clinical history, symptoms, and physical examinations were often ignored in these studies. Critical values of acute-phase reactants typically associated with infections, including the C-reactive protein and procalcitonin, are also used to predict mortality of patients with general sepsis, with a moderate predictive power [10]. However, the predictive capacity of these biomarkers in patients with Kp-BSI remains unknown. As Kp-BSI frequently leads to sepsis, the Sequential Organ Failure Assessment (SOFA) score is accepted as an indicator of mortality in patients with Kp-BSI [11]. However, few studies have validated the sensitivity and specificity of the SOFA score in predicting the mortality of patients with Kp-BSI; moreover, the organ-based SOFA sub-scores are not specific for *K. pneumoniae* infections. In pediatric patients with sepsis, the age-adapted SOFA score is a promising prognostic instrument [12], but it has not been validated in children with a specific type of infection. Overall, risk factors and early predictors of mortality in children with Kp-BSI have not been thoroughly investigated.

In this study, we aimed to identify prognostic predictors of mortality and evaluate whether consecutive combination of pediatric SOFA score with clinical characteristics or biomarkers could improve the score’s ability to predict mortality in a cohort of pediatric patients with Kp-BSI.

## Methods

### Study design

This was a 10-year retrospective cohort study conducted at Xinhua Hospital affiliated to Shanghai Jiao Tong University School of Medicine, a 3000-bed tertiary care university teaching hospital in Shanghai, China. Pediatric patients (≤ 18 years old) diagnosed with Kp-BSI between January 2009 and June 2019 were included in the study. Patients who were transferred from other hospitals were excluded. The primary outcome was the 90-day mortality after diagnosis of Kp-BSI; the 14- and 30-day mortality were also recorded. Discharged patients made routine visits to the clinic every month. Patient follow-up was performed retrospectively by phone interviews and by checking medical records. The study was approved by the ethics committee and the review board of Xinhua Hospital (ID: XHEC-C-2019-109) and complied with the ethical principles stated by the Declaration of Helsinki.

### Clinical data collection

All medical records of the enrolled patients were reviewed by a team of physicians from the microbiology department, the infectious diseases department, and the pediatric/neonatal intensive care unit. The following variables were collected from the medical charts: demographic characteristics (age, sex, admission year, and admission ward), birth status (preterm birth and cesarean delivery), the weight-for-age z-score (calculated using the Anthro software version 3.2.2) [13], comorbidities (congenital gastrointestinal anomalies, congenital heart diseases, and malignancies), probable source of infection, prior hospitalization in the last 6 months, invasive treatments before the onset of Kp-BSI (surgery, urinary catheterization, gastrointestinal decompression, sputum suction, and invasive ventilation), and levels of biomarkers (leukocyte counts, platelet counts, hemoglobin, procalcitonin, C-reactive protein, albumin, total bilirubin, creatinine, and lactate). The severity of organ failure was measured using SOFA score age-adapted for children [12]. The level of biomarkers and risk scores at the time of Kp-BSI onset were determined within 24 h after the index blood culture [14]. The definitive antimicrobial treatments, which defined as the agents used for the longest duration within the first week after the index blood culture [15], were recorded for children with CR-Kp bloodstream infection.

### Definitions and methods of microbiology

A case of bloodstream infection was defined as positive blood cultures from two separate venipuncture sites. A solitary blood culture was considered contaminated and excluded from the analysis [16]. Bloodstream infections were considered healthcare-associated in patients who stayed in the hospital for more than 48 h or fulfilled other published criteria [17]. *K. pneumoniae* isolates were identified by matrix-assisted laser desorption ionization time-of-flight mass spectrometry (MALDI-TOF MS, Bruker Daltonics, Bremen, Germany). The antibiotic susceptibilities of the isolates were determined using Vitek 2 automated system (BioMérieux, Marcy l’ Etoile, France), combined with a disk diffusion method. The results were interpreted according to the Clinical and Laboratory Standards Institute criteria [18]. Isolates resistant to at least one carbapenem were considered carbapenem-resistant. Polymicrobial bloodstream infections were defined as the isolation of more than one bacterial species from a set of blood culture bottles [19]. Intra-abdominal infection was broadly defined as localized or diffuse peritoneal inflammation caused by microorganisms. The source of the bloodstream infection and the specific infection sites were determined by two investigators according to the standard definitions by the Centers for Disease Control and Prevention [20].

### Statistical analysis

Continuous variables with normal distribution were presented as mean ± standard deviation; non-normal variables were presented as median and interquartile range; and categorical variables were described as frequencies. Differences in continuous variables between survivors and non-survivors were compared using the Student’s *t*-test or the nonparametric Mann-Whitney *U* test. Proportions were compared using the χ-squared test for large samples, and the Fisher’s exact probability test for small samples. To estimate the association between patients’ clinical characteristics and mortality, we evaluated the hazard ratio (HR) and the 95% confidence interval (CI) using the Cox proportional hazard model. We grouped the levels of procalcitonin, C-reactive protein, and albumin within the interquartile range (between P25 and P75) for the regression analysis. We used a multivariate Cox regression adjusted for baseline characteristics (age, sex, admission year, and admission ward) to identify independent risk factors of mortality. Risk factors with *P* <  0.10 in the Cox analysis were introduced stepwise into the predictive model as additional components of the SOFA score. Receiver operating characteristic curves were analyzed and each respective area under the curve (AUC) was calculated to evaluate the discriminatory power of single parameters and combined models for prediction of mortality.

To quantify the incremental value of the modified SOFA scores, we compared the predictive performance of the models using the DeLong’s test [21] and the integrated discrimination improvement index [22]. An optimal score (Kp-specific SOFA) was established using the model reported by Sullivan et al. [23]. Three cut-off points of the risk score were selected to stratify patients at three-tiered risk corresponded to the observed mortality rates. The Kaplan-Meier curves and log-rank tests were used to compare survival between risk-stratified patients. A two-tailed *P*-value of less than 0.05 was considered statistically significant. The R software version 3.6.1 (http://www.R-project.org) and the EmpowerStats (www.empowerstats.com; X&Y Solutions, Inc., Boston, MA, USA) were used for all statistical analyses.

## Results

### Characteristics of the study population

During the study period (January 2009 to June 2019) there were 50,000 estimated admissions per year in pediatric wards at Xinhua Hospital, and 154 (0.03%) patients were diagnosed with Kp-BSI. Eight (5.5%) patients were lost to follow-up; a total of 146 patients were included in this retrospective study. Overall, 20 (13.7%), 25 (17.1%), and 33 (22.6%) patients died within 14, 30, and 90 days from Kp-BSI onset, respectively.

The median age of patients was 2.7 months (interquartile range: 1.0, 10.9 months, Table 1). Bloodstream infections were mainly secondary to pneumonia (50.0%), followed by intra-abdominal infection (20.5%) and indwelling central catheters (6.8%). Primary bacteremia occurred in 22.6% of patients (Table 2). Most of the Kp-BSI (84.9%) cases were healthcare-associated, and nearly half of the patients (48.6%) were infected with CR-Kp. The most common organ failure within 24 h from Kp-BSI onset was respiratory failure (63.0%), followed by hepatic failure (52.7%), coagulation disorder (39.7%), shock/hypotension (29.5%), renal failure (22.6%), and altered mental status (15.1%). Only 11.6% of the patients had no organ failure at the time of Kp-BSI onset (Table 2).

**Table 1:** Baseline characteristics of the survivors and the non-survivors in children with *Klebsiella pneumoniae* bloodstream infection during January 2009 to June 2019

Variable	Total (***N*** = 146)	Survivors (***n*** = 113)	Non-survivors (***n*** = 33)	***P*** value
Boys, n (%)	89 (61.0)	69 (61.1)	20 (60.6)	0.96
Age, months, median (IQR)	2.7 (1.0, 10.9)	2.3 (0.8, 10.4)	4.0 (1.3, 14.7)	0.13
Age group				0.38
≤ 1 month, n (%)	38 (26.0)	32 (28.3)	6 (18.2)	
1–12 months, n (%)	74 (50.7)	57 (50.4)	17 (51.5)	
> 12 months, n (%)	34 (23.3)	24 (21.2)	10 (30.3)	
Weight-for-age z-score, median (IQR)	−0.7 (− 1.8, 0)	−0.7 (− 1.8, 0)	−0.5 (− 2.5, 0.4)	0.65
Preterm birth, n (%)	48 (32.9)	33 (29.2)	15 (45.5)	0.08
Cesarean delivery, n (%)	81 (55.5)	64 (56.6)	17 (51.5)	0.60
Prior hospitalization within 6 months, n (%)	66 (45.2)	44 (38.9)	22 (66.7)	**< 0.01**
Underlying comorbidities				
Congenital gastrointestinal anomalies^a^, n (%)	61 (41.8)	48 (42.5)	13 (39.4)	0.75
Congenital heart disease, n (%)	55 (37.7)	44 (38.9)	11 (33.3)	0.56
Malignancies, n (%)	11 (7.5)	6 (5.3)	5 (15.2)	0.13
Admission year				0.43
2009–2012, n (%)	28 (19.2)	22 (19.5)	6 (18.2)	
2013–2016, n (%)	43 (29.5)	36 (31.9)	7 (21.2)	
2017–2019, n (%)	75 (51.4)	55 (48.7)	20 (60.6)	
Admission ward				0.20
Pediatric intensive care unit, n (%)	18 (12.3)	11 (9.7)	7 (21.2)	
Pediatric surgery intensive care unit, n (%)	49 (33.6)	38 (33.6)	11 (33.3)	
Neonatal intensive care unit, n (%)	36 (24.7)	27 (23.9)	9 (27.3)	
Others, n (%)	43 (29.5)	37 (32.7)	6 (18.2)	

*IQR* interquartile range

^a^Congenital gastrointestinal anomalies included esophageal atresia, intestinal atresia, anal atresia, and diaphragmatic hernia

**Table 2:** Mortality predictors at the onset of *Klebsiella pneumoniae* bloodstream infection

Variable	Total (***N*** = 146)	Survivors (***n*** = 113)	Non-survivors (***n*** = 33)	***P*** value
**Invasive procedures prior to the onset of infection**				
Surgery, n (%)	66 (45.2)	52 (46.0)	14 (42.4)	0.72
Urinary catheterization, n (%)	72 (49.3)	57 (50.4)	15 (45.5)	0.61
Gastrointestinal decompression, n (%)	85 (58.2)	66 (58.4)	19 (57.6)	0.93
Sputum suction, n (%)	67 (45.9)	52 (46.0)	15 (45.5)	0.95
Invasive ventilation, n (%)	54 (37.0)	40 (35.4)	14 (42.4)	0.46
**Source of bloodstream infection**				
Primary bacteremia, n (%)	33 (22.6)	30 (26.5)	3 (9.1)	**0.04**
Intra-abdominal infection, n (%)	30 (20.5)	14 (12.4)	16 (48.5)	**< 0.01**
Pneumonia, n (%)	73 (50.0)	60 (53.1)	13 (39.4)	0.17
Catheter-related infection, n (%)	10 (6.8)	9 (8.0)	1 (3.0)	0.46
**Microbiological characteristics**				
Healthcare-associated infection, n (%)	124 (84.9)	96 (85.0)	28 (84.8)	0.83
Carbapenem-resistance isolate, n (%)	71 (48.6)	50 (44.2)	21 (63.6)	0.05
Polymicrobial bloodstream infection, n (%)	34 (23.3)	26 (23.0)	8 (24.2)	0.88
**Laboratory predictors**^**a**^				
Leukocyte counts, median (IQR**)**, 10^9^/L	9.9 (5.3, 18.9)	9.3 (6.7, 15.7)	13.1 (5.0, 24.5)	0.44
Hemoglobin, median (IQR**)**, g/L	105 (89, 121)	105 (93, 122)	97 (86, 118)	0.84
Platelet counts, median (IQR**)**, 10^9^/L	190 (102, 282)	213 (115, 314)	103 (17, 151)	**< 0.01**
Procalcitonin, median (IQR**)**, ng/mL	1.4 (0.1, 17.0)	0.8 (0, 13.8)	7.5 (0.32, 69.6)	0.06
C-reactive protein, median (IQR**)**, mg/L	30 (8, 77)	19 (8, 54)	88 (39, 143)	**< 0.01**
Albumin, median (IQR**)**, g/L	28.0 (27.6, 35.4)	32.4 (28.8, 35.8)	26.8 (20.1, 34.0)	**< 0.01**
Total bilirubin, median (IQR**)**, mg/dL	1.6 (0.5, 3.9)	1.1 (0.4, 3.3)	2.2 (1.3, 5.8)	0.06
Creatinine, median (IQR**)**, μmol/L	26 (21, 43)	25 (20, 37)	40 (22, 64)	**0.01**
Lactate, median (IQR), mmol/L	1.6 (0.8, 4.1)	1.4 (0.8, 2.4)	2.4 (1.4, 5.3)	**< 0.01**
**Organ failure**				
SOFA score, median (IQR)	5 (3, 7)	4 (2, 6)	7 (6, 10)	**< 0.01**
Altered mental status, n (%)	22 (15.1)	10 (8.9)	12 (36.4)	**< 0.01**
Respiratory failure, n (%)	92 (63.0)	68 (60.2)	24 (72.7)	0.19
Shock/hypotension, n (%)	43 (29.5)	28 (24.8)	15 (45.5)	**0.02**
Hepatic failure, n (%)	77 (52.7)	53 (46.9)	24 (72.7)	**< 0.01**
Renal failure, n (%)	33 (22.6)	22 (19.5)	11 (33.3)	0.09
Coagulation disorder, n (%)	58 (39.7)	35 (31.0)	23 (69.7)	**< 0.01**
Number of organ failure(s)				**< 0.01**
0, n (%)	17 (11.6)	15 (13.3)	2 (6.1)	
1, n (%)	36 (24.7)	34 (43.4)	2 (6.1)	
2, n (%)	35 (24.0)	30 (26.5)	5 (15.2)	
3, n (%)	28 (19.2)	17 (15.0)	11 (33.3)	
≥ 4, n (%)	30 (20.5)	17 (15.0)	13 (39.3)	

*IQR* interquartile range, *SOFA* sequential organ failure assessment

^a^Laboratory examinations were performed within 24 h after the collection of blood culture samples

### Risk factors associated with 90-day mortality in patients with Kp-BSI

**Fig. 1:**
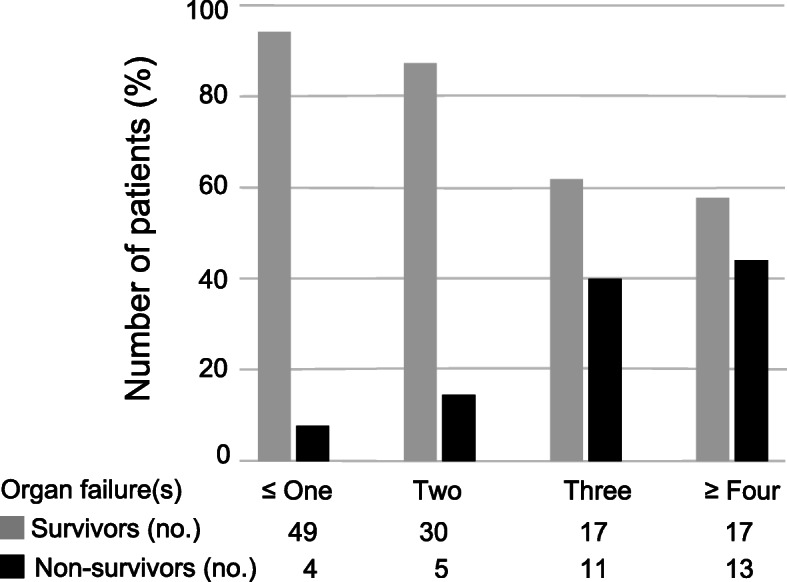
Ninety-day mortality and survivor numbers according to the number of organ failure(s) in children with *Klebsiella pneumoniae* bloodstream infection

A total of 33 (22.6%) patients died within 90 days after the onset of Kp-BSI, and 113 (77.4%) were discharged from the hospital after reaching clinical stability. No significant differences in baseline characteristics (age, sex, admission year, and admission ward, weight-for-age z-score) were observed between survivors and non-survivors (Table 1). There were more patients with a history of preterm birth and a malignant disease as comorbidity among the non-survivor group compared to the survivor group. Non-survivors were characterized by a history of hospitalization in the last 6 months (*P* <  0.01, Table 1). Before the onset of Kp-BSI, no differences in invasive procedures during hospitalization were observed between survivors and non-survivors (Table 2). Intra-abdominal sources of infection were more frequent among non-survivors (48.5%) than survivors (12.4%), while bloodstream infections of patients alive at day 90 were more likely to result from primary bacteremia. The prevalence of carbapenem-resistant strains in the survivors and the non-survivors was 44.2 and 63.6%, respectively. Compared to the survivors, the non-survivors had lower levels of serum albumin and platelet counts, and higher levels of C-reactive protein, procalcitonin, total bilirubin, creatinine, and lactate. Additionally, the median SOFA score of non-survivors was approximately 2-fold higher than that of survivors. The increased number of organ failures at the onset of Kp-BSI was associated with a higher risk of mortality (Fig. 1). The proportion of non-survivors with abnormal function of at least three organ systems was 41.4% (24/58).

After adjusting for age, sex, admission year and admission ward, patients were nearly twice as likely to die if they were born prematurely, and prior hospitalization in the last 6 months was associated with a 2.4-fold increase in mortality (Table 3). For patients infected with *K. pneumoniae* from intra-abdominal sources, the risk of death was 6.8-fold higher compared to that of patients with primary bacteremia or catheter-related infections. Malignancies, carbapenem-resistant isolates, and respiratory failure were not significantly associated with mortality. Among the various types of organ failure, altered mental status was associated with the worst outcome (HR = 5.19, 95% CI 2.50–10.77), followed by coagulation disorder (HR = 4.54, 95% CI 2.12–9.72), hepatic failure (HR = 2.90, 95% CI 1.33–6.35), shock/hypotension (HR = 2.41, 95% CI 1.21–4.82), and renal failure (HR = 2.20, 95% CI 1.02–4.73). Low levels of albumin and high levels of sepsis-related biomarkers, including the C-reactive protein and lactate, were also significant risk factors for mortality (Table 3).

**Table 3:** Multivariate Cox proportional hazard regression results for risk factors of the 90-day mortality

Predictor		No. of deaths/total (%)	Adjusted HR^**a**^ (95% CI)	***P*** value
Preterm birth		15/48 (31.3)	2.25 (1.04, 4.86)	**0.04**
Malignancies		5/11 (45.5)	2.36 (0.74, 7.56)	0.15
Prior hospitalization within 6 months		22/66 (33.3)	2.40 (1.12, 5.14)	**0.02**
Carbapenem-resistant isolate		21/71 (29.6)	2.13 (0.98, 4.61)	0.06
**Source of bloodstream infection**				
Primary or catheter-related		4/43 (9.3)	Ref	–
Pneumonia		13/73 (17.8)	1.69 (0.51, 5.58)	0.39
Intra-abdominal infection		16/30 (53.3)	6.75 (2.11, 21.57)	**< 0.01**
**Organ failure**				
Altered mental status		12/22 (54.5)	5.19 (2.50, 10.77)	**< 0.01**
Respiratory failure		24/92 (26.1)	1.40 (0.61, 3.20)	0.42
Shock/hypotension		15/43 (34.9)	2.41 (1.21, 4.82)	**0.01**
Hepatic failure		24/77 (31.2)	2.90 (1.33, 6.35)	**< 0.01**
Renal Failure		11/33 (33.3)	2.20 (1.02, 4.73)	**0.04**
Coagulation disorder		23/58 (39.7)	4.54 (2.12, 9.72)	**< 0.01**
**Laboratory predictor**^**b**^				
Procalcitonin (ng/mL)	< 0.1	7/43 (16.3)	Ref	
	0.1–17	15/65 (23.1)	1.17 (0.43, 3.19)	0.76
	> 17	11/38 (28.9)	1.72 (0.60, 4.91)	0.31
C-reactive protein (mg/L)	< 8	5/47 (10.6)	Ref	
	8–77	9/60 (15)	1.48 (0.48, 4.53)	0.49
	> 77	19/39 (48.7)	6.46 (2.26, 18.52)	**< 0.01**
Albumin (g/L)	> 35	6/40 (15.0)	Ref	
	28–35	8/58 (13.8)	0.91 (0.32, 2.63)	0.86
	< 28	19/48 (39.6)	3.32 (1.31, 8.40)	**0.01**
Lactate (mmol/L)	< 2	17/101 (16.8)	Ref	
	≥ 2	16/45 (35.6)	3.44 (1.46, 8.11)	**< 0.01**

*HR* hazard ratio

^a^Multivariate adjustment was made for age, sex, admission ward and admission year

^b^Procalcitonin, C-reactive protein and albumin were categorized into three strata by the boundaries of interquartile range (P25 and P75)

### Antimicrobial treatments in patients infected with CR-Kp

Among the 71 patients with CR-Kp bloodstream infection, 40 (56.3%) received a carbapenem combined with another antibiotic as a definitive therapy, proven to be effective in vitro (including amikacin, fosfomycin, or polymyxin B), while the remainder received a high-dose extended or continuous infusion of a carbapenem. The mortality rate of patients receiving polymyxin B plus carbapenem or fosfomycin plus carbapenem was 19.4% (6/31), while that of patients treated with amikacin plus carbapenem and with carbapenem monotherapy was 44.4% (4/9) and 35.5% (11/31), respectively [see Table S1, Additional file 1]. Additionally, a subgroup analysis using Cox proportional hazards model showed no significant association between the type of definitive antimicrobial therapy and the 90-day mortality of patients with CR-Kp bloodstream infection [see Table S1, Additional file 1].

### Development of a combined model on the basis of the SOFA score

**Fig. 2:**
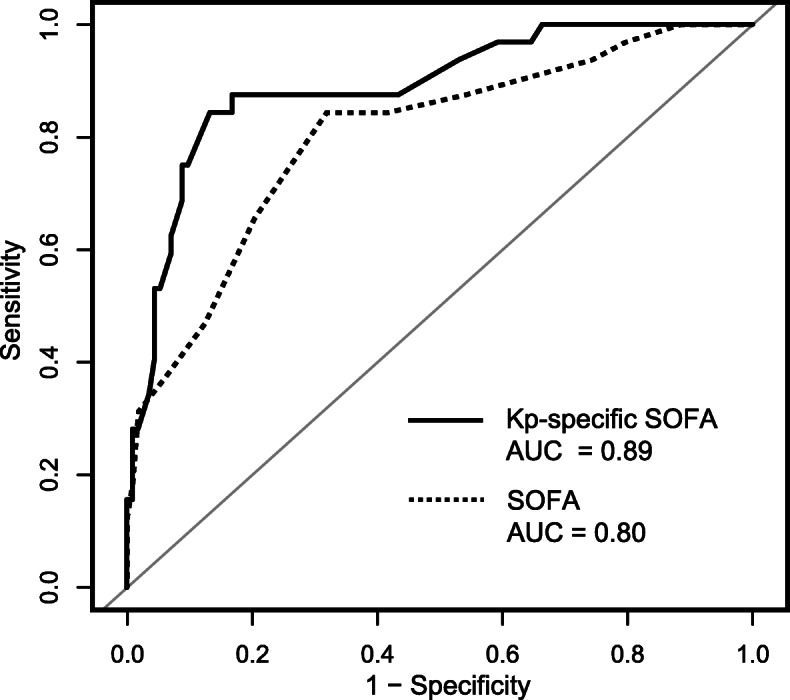
Receiver operating characteristic curves of the original SOFA score and the Kp-specific SOFA score for predicting 90-day mortality. The Kp-specific SOFA score was developed by multivariate regression (Logit [mortality] = − 5.86 + 0.53 × [SOFA score] + 1.81 × [prior hospitalization, 0 or 1] + 1.83 × [intra-abdominal source, 0 or 1]). The difference in AUC values between the two models was significant according to the DeLong’s test (*P* < 0.01)

The pediatric SOFA score had a moderate predictive power for 90-day mortality, with an AUC of 0.80 (95% CI 0.71–0.89), along with a high sensitivity (0.84) and a low specificity (0.68). The predictive value of clinical characteristics and blood biomarkers was also evaluated. Each combination of SOFA with risk factors (including prior hospitalization, intra-abdominal source of infection, C-reactive protein, and albumin) improved the predictive performance of the model to different degrees [see Table S2, Additional file 1]. A modified SOFA model combining SOFA plus prior hospitalization and intra-abdominal source of infection was selected as the optimal multivariate predictive model (AUC = 0.89, 95% CI 0.82–0.96; sensitivity = 0.86; specificity = 0.84; positive predictive value = 0.64; negative predictive value = 0.95), outperforming the original SOFA score (DeLong’s test, *P* <  0.01, Fig. 2). According to the integrated discrimination improvement index, other combinations of SOFA with risk factors had no additional prognostic value over the selected model [see Table S3, Additional file 1]. The final multivariate model (Logit [mortality] = − 5.86 + 0.53 × [SOFA score] + 1.81 × [prior hospitalization, 0 or 1] + 1.83 × [intra-abdominal source, 0 or 1]) was used to develop a scoring system (Kp-specific SOFA) including: SOFA (original score), prior hospitalization (4 points) and intra-abdominal source (4 points). The mean estimated risk score for the study population was 8 ± 4 (range 0–21).

**Fig. 3:**
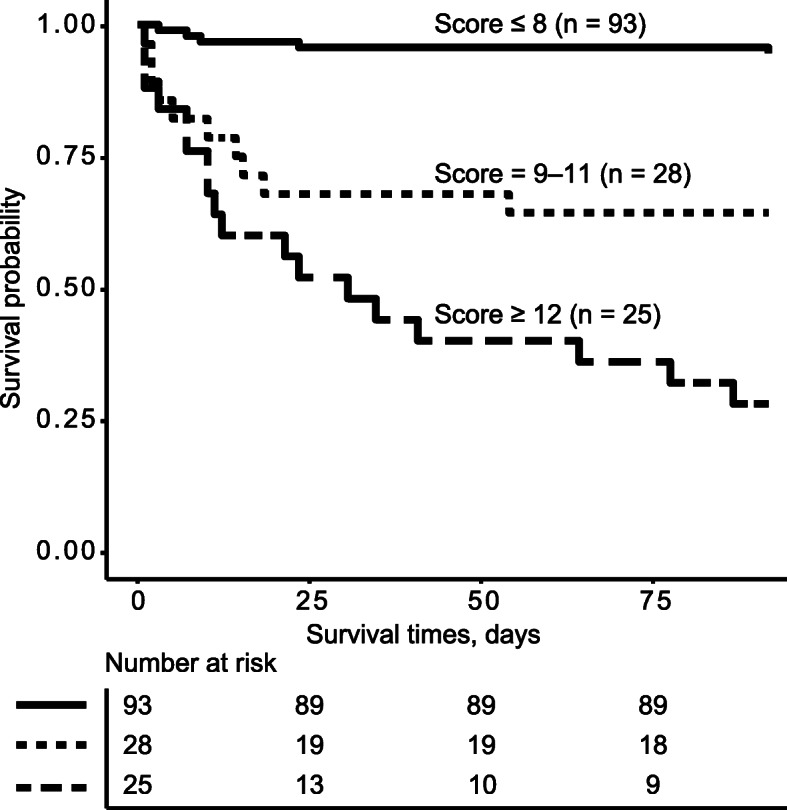
Ninety-day survival probabilities at three risk categories (score ≤ 8, 9–11 and ≥ 12) after the onset of *Klebsiella pneumoniae* bloodstream infection in children estimated by the Kaplan-Meier analysis (log-rank test, *P* < 0.01)

The observed risk of mortality and the estimated probabilities based on the Kp-specific SOFA for three risk strata (low, medium, and high) are shown in Table 4. The 90-day mortality rate was 5.4%, 35.7%, and 72.0% for low- (score 0–8), medium- (score 9–11), and high-risk (score ≥ 12) patients, respectively. Compared to the low-risk group, patients with medium- or high-risk scores were more likely to die (HR = 8.36, 95% CI 3.60–27.83 for medium-risk and HR = 20.27, 95% CI 7.47–54.95 for high-risk; Table 4). This Kp-specific SOFA score was able to stratify patients into low-, medium-, and high-risk groups on the basis of the Kaplan-Meier survival curves (log-rank test, *P* <  0.01, Fig. 3).

**Table 4:** Risk categories for the 90-day mortality after the onset of *Klebsiella pneumoniae* bloodstream infection

Risk category	Estimated risk scores	Estimated risk of mortality (%)	No. of non-survivors/total no. of patients (%)	Cox Analysis	
				HR (95% CI)	***P*** value
Low risk	≤ 8	≤16.24	5/93 (5.4)	Ref	–
Medium risk	9–11	24.73–48.55	10/28 (35.7)	8.36 (3.60, 27.83)	< 0.01
High risk	≥ 12	≥ 61.53	18/25 (72.0)	20.27 (7.47, 54.95)	< 0.01

*HR* hazard ratio

### Kp-specific SOFA in predicting 30-day mortality

The 30-day mortality rate was 17.1% (25/146). For predicting 30-day mortality, the AUC value of pediatric SOFA and Kp-specific SOFA was 0.79 and 0.85, respectively [see Figure S1, Additional file 1]. Three categories of risk were performed by Kaplan-Meier survival curves using the predetermined cut-off values, which demonstrated a significant difference in 30-day mortality among three strata by log-rank test (*P* <  0.01) [see Figure S2, Additional file 1].

## Discussion

In this 10-year retrospective study, we aimed to investigate the early predictors of mortality in patients with Kp-BSI. We found that a history of preterm birth, hospitalization in the last six months, intra-abdominal source of infection, organ failure, low serum albumin, and high levels of C-reactive protein and lactate, were independent risk factors for 90-day mortality. In particular, we demonstrate for the first time that combining two risk factors (prior history of hospitalization and intra-abdominal source of infection) with the pediatric SOFA score at the early onset of Kp-BSI has a satisfactory predictive power of 30- and 90-day mortality, with an AUC value of 0.85 and 0.89, respectively. Adding these two variables raised the score’s specificity from 0.68 to 0.84 for predicting 90-day mortality. Moreover, this Kp-specific SOFA was also able to group patients by three mortality strata.

In recent years, the global prevalence of *K. pneumoniae* has increased rapidly. A multi-center study in the US reported a 3.8% point prevalence of *K. pneumoniae* [24]. In China, the prevalence of *K. pneumoniae* was found to be 12.0% among 244,843 clinical isolates, of which 19.1% were collected from the bloodstream [25]. The overall mortality reported in the literature for adult patients was as high as 29.3% [26]. In our pediatric study, the 90-day mortality was 22.6%, which was higher than that reported previously in a tertiary children’s hospital in China (12.3%) [6]. The common presence of multiple organ failure at the onset of Kp-BSI in our study population might explain the high mortality rate. As *K. pneumoniae* is able to evade early innate immune reactions, it can cause systemic toxicity and result in multi-organ failure [27]. Septic shock and mechanical ventilation at the early stages of Kp-BSI are considered strong risk factors for mortality [6, 28]. In our study, the respiratory tract was the most common organ to fail at the onset of Kp-BSI, but this was not associated with fatal outcomes. Altered mental status, coagulation disorder, hepatic failure, shock/hypotension, and renal failure were significantly associated with mortality. We demonstrated that the risk of mortality increased with high SOFA scores at Kp-BSI onset, thereby highlighting the importance of systematic evaluations of clinical parameters relevant to organ function at the early stages of infection.

Apart from organ failure, there was a significant difference in mortality between patients with- and patients without- prior hospitalization in the last six months, though significant difference in the specific categories of comorbidity was not observed. Impaired host defense in children with severe underlying conditions is responsible for severe sepsis and poor prognosis [29]. Additionally, complicated intra-abdominal infections, including necrotizing enterocolitis, bacterial peritonitis, and intra-abdominal sepsis associated with bowel perforation, are serious complications in premature babies and children recovering from surgery or with other serious conditions. The gastrointestinal system is considered critical for the host resistance to sepsis, with the bacterial translocation and microbiota disturbances in critically ill patients supporting the concept of impaired communication across the gut-organ axes [30, 31]. Moreover, tissue damage caused by intra-abdominal sepsis can be a source of pro-inflammatory cytokines that induce distant organ failure [32]. Compromised gut-barrier functions also perturb the clearance of bacteria and endotoxins from the systemic circulation [33]. In this study, the intra-abdominal source of bloodstream infection was associated with the worst prognosis. However, the impact of the primary infection site on mortality in patients with Kp-BSI is controversial and needs to be investigated further [34, 35].

Additionally, we explored several biomarkers that have not been considered for SOFA sub-scores but have been commonly measured in the intensive care unit. The combination of serum albumin and C-reactive protein levels with SOFA also improved risk prediction of mortality. Albumin, which is exclusively synthesized by the liver and reflects the physiological response to injury and infection, has been identified as an independent predictor of mortality in patients with Kp-BSI [36]. On the other hand, C-reactive protein is an acute-phase reactant with prognostic significance in patients with infections [10], and its response to infection is determined by the invasive mechanisms as well as host predisposition [37]. Although the measurement of multiple biomarkers may improve the identification of patients at risk, it may increase the complexity and costs of the diagnostic processes. Moreover, including the levels of C-reactive protein and albumin in the risk model added only a minimal advantage compared to the Kp-specific SOFA, as determined by the integrated discrimination improvement analysis.

In this study, we validated the pediatric SOFA score updated in 2017 to prognosticate the outcomes of children with Kp-BSI, and determined its AUC value to be 0.80. This finding filled the knowledge gap on the prognostic value of SOFA when applied to specific infections. Based on this data, and to address the urgent need for research on prognostic factors in pediatric Kp-BSI, we developed a Kp-specific SOFA score with enhanced AUC value by adding two risk factors as sub-scores. The Kp-specific SOFA had a high negative predictive value (0.95), suggesting that if extensively applied in the clinical field, it may be a reasonable reference to rule out high-risk pediatric patients. Most of our study population consisted of patients with healthcare-associated infection and with different comorbidities, which was representative of children with Kp-BSI in clinical practice. Therefore, our results can be extrapolated to the pediatric population with Kp-BSI. Considering different etiologies beyond Kp-BSI, combination of risk factors with the SOFA score also may strengthen its predictive accuracy in identification of high-risk patients related to other sites of infection and with infections caused by other pathogens [38].

This study had some limitations. First, this was a retrospective study and the recall bias might be a problem. Prospective validation was not conducted for the risk score derived from our study population. Nevertheless, the recall bias has been reduced as all 146 patients underwent regular follow-ups in this hospital, which included electronic medical records, for 90 days after the onset of Kp-BSI. Second, 48.6% of the children were infected by CR-Kp and received treatments including extended/continuous infusion of carbapenem and carbapenem plus amikacin, fosfomycin, or polymyxin B. However, it is generally accepted that the first-line treatment of CR-Kp infection is based on colistin or a novel cephalosporin/β-lactamase inhibitor combination, ceftazidime-avibactam [39], which was not used in this study group. Considering the paucity of available data in pediatric population [40], the therapy used at our institution was adjusted on the basis of the severity of infection, the susceptibility profile of isolates, and pharmacokinetics to achieve adequate antibiotic serum concentrations. Treatment is a crucial part for patient prognosis, but we did not consider the antimicrobial therapy as a factor in our predictive model due to the inconsistency between the treatments in this retrospective cohort and the current standard. Third, this was a single-center study with a potential selection bias. Future multicenter studies are needed to better determine the clinical value of the Kp-specific SOFA at the early stages of Kp-BSI in children.

## Conclusion

In conclusion, hospitalization in the six months, intra-abdominal source of infection and organ failure at the early onset of Kp-BSI were important factors determining the clinical outcomes of pediatric patients. The Kp-specific SOFA may offer a better predictive tool of 90-day mortality in children with Kp-BSI.

## Supplementary Information

**Additional file 1: Table S1.** Antimicrobial therapy and 90-day mortality in 71 patients with bloodstream infection caused by carbapenem-resistant *Klebsiella pneumoniae*. In patients with CR-Kp bloodstream infection, there was no significant association between the types of definitive antimicrobial therapy and mortality according to Cox proportional hazards model. **Table S2.** Predictive performance of the single predictors and the combined models for predicting 90-day mortality. The SOFA score achieved the best predictive performance among single predictors. Any combination of SOFA with risk factors (prior hospitalization, intra-abdominal source of infection, levels C-reactive protein, and albumin) improved the predictive performance. **Table S3.** The added prognostic value of the predictive models as determined by the integrated discrimination improvement index. The combinations of other predictors had no additional prognostic value over the final model: SOFA + prior hospitalization + intra-abdominal source of infection. **Figure S1.** Receiver operating characteristic curves of the original SOFA score and the Kp-specific SOFA score for predicting 30-day mortality. **Figure S2.** Thirty-day survival probabilities at three risk categories (score ≤ 8, 9–11 and ≥ 12) after the onset of *Klebsiella pneumoniae* bloodstream infection in children estimated by the Kaplan-Meier analysis (log-rank test, *P* <  0.01)

## Data Availability

The datasets analyzed during the current study are available from the corresponding author on reasonable request.

## Abbreviations

AUC: Area under the curve
CI: Confidence interval
CR-Kp: Carbapenem-resistant *Klebsiella pneumoniae*
HR: Hazard ratio
Kp-BSI: *Klebsiella pneumoniae* bloodstream infection
SOFA: Sequential Organ Failure Assessment
