# Supplementary material for: A risk score based on pediatric sequential organ failure assessment predicts 90-day mortality in children with Klebsiella pneumoniae bloodstream infection
Source: BMC Infect Dis. 2020 Dec 2;20:916. doi: 10.1186/s12879-020-05644-w (PMC7709332; doi:10.1186/s12879-020-05644-w)
Supplement: Supplementary file 1 — Additional file 1: Table S1. Antimicrobial therapy and 90-day mortality in 71 patients with bloodstream infection caused by carbapenem-resistant Klebsiella pneumoniae. In patients with CR-Kp bloodstream infection, there was no significant association between the types of definitive antimicrobial therapy and mortality according to Cox proportional hazards model. Table S2. Predictive performance of the single predictors and the combined models for predicting 90-day mortality. The SOFA score achieved the best predictive performance among single predictors. Any combination of SOFA with risk factors (prior hospitalization, intra-abdominal source of infection, levels C-reactive protein, and albumin) improved the predictive performance. Table S3. The added prognostic value of the predictive models as determined by the integrated discrimination improvement index. The combinations of other predictors had no additional prognostic value over the final model: SOFA + prior hospitalization + intra-abdominal source of infection. Figure S1. Receiver operating characteristic curves of the original SOFA score and the Kp-specific SOFA score for predicting 30-day mortality. Figure S2. Thirty-day survival probabilities at three risk categories (score ≤ 8, 9–11 and ≥ 12) after the onset of Klebsiella pneumoniae bloodstream infection in children estimated by the Kaplan-Meier analysis (log-rank test, P < 0.01) [file 12879_2020_5644_MOESM1_ESM.docx]

**Table S1. Antimicrobial therapy and 90-day mortality in 71 patients with bloodstream infection caused by carbapenem-resistant *Klebsiella pneumoniae***

| **Definitive antimicrobial therapy** | **No. of deaths**  **/Total (%)** | **Hazard Ratio**  **(95% CI)** | ***P* value** |
| --- | --- | --- | --- |
| **Type of therapy** |  |  |  |
| High-dose extended/continuous infusion  of carbapenem monotherapy | 11/31 (35.5) | Ref | - |
| Amikacin + carbapenem | 4/9 (44.4) | 1.20 (0.38, 3.80) | 0.76 |
| Fosfomycin + carbapenem | 4/22 (18.2) | 0.51 (0.16, 1.60) | 0.25 |
| Polymyxin B + carbapenem | 2/9 (22.2) | 0.74 (0.16, 3.40) | 0.70 |
| **Polymyxin B/fosfomycin based therapy vs. other therapies** | | | |
| Polymyxin B/fosfomycin + carbapenem | 6/31 (19.4) | Ref | - |
| Amikacin + carbapenem | 4/9 (44.4) | 2.11 (0.59, 7.58) | 0.25 |
| Carbapenem monotherapy | 11/31 (35.5) | 1.76 (0.65, 4.79) | 0.26 |
| **Combination therapy vs. carbapenem monotherapy** | | | |
| Combination therapy | 10/40 (25.0) | Ref | - |
| Carbapenem monotherapy | 11/31 (35.5) | 1.39 (0.59, 3.29) | 0.45 |

**Table S2. Predictive performance of the single predictors and the combined models for predicting 90-day mortality**

| **Predictive model** | **AUC (95% CI)** | **Sensitivity** | **Specificity** | **Accuracy** | **PPV** | **NPV** |
| --- | --- | --- | --- | --- | --- | --- |
| **Single predictors** | | | | | | |
| Prior hospitalization within 6 months ① | 0.64 (0.55, 0.73) | 0.67 | 0.61 | 0.62 | 0.33 | 0.86 |
| Intra-abdominal source ② | 0.68 (0.59, 0.77) | 0.67 | 0.88 | 0.79 | 0.53 | 0.85 |
| Premature ③ | 0.58 (0.49, 0.68) | 0.71 | 0.45 | 0.65 | 0.31 | 0.82 |
| Carbapenem-resistant isolate ④ | 0.60 (0.50, 0.69) | 0.64 | 0.56 | 0.58 | 0.30 | 0.84 |
| C-reactive protein (stratified) ⑤ | 0.72 (0.62, 0.82) | 0.58 | 0.82 | 0.77 | 0.49 | 0.87 |
| Albumin (stratified) ⑥ | 0.66 (0.55, 0.76) | 0.58 | 0.74 | 0.71 | 0.40 | 0.86 |
| Lactate (stratified) ⑦ | 0.61 (0.52, 0.71) | 0.48 | 0.74 | 0.68 | 0.36 | 0.83 |
| SOFA score ⑧ | 0.80 (0.71, 0.89) | 0.84 | 0.68 | 0.72 | 0.43 | 0.94 |
| **Adding predictors to the SOFA score** | | | | | | |
| SOFA + Prior hospitalization ① | **0.86 (0.79, 0.93)** | 0.78 | 0.80 | 0.79 | 0.52 | 0.92 |
| SOFA + Intra-abdominal source ② | **0.84 (0.76, 0.93)** | 0.59 | 0.96 | 0.88 | 0.79 | 0.89 |
| SOFA + Premature ③ | 0.80 (0.71, 0.89) | 0.84 | 0.68 | 0.72 | 0.43 | 0.94 |
| SOFA + Carbapenem-resistant isolate ④ | 0.81 (0.73, 0.90) | 0.81 | 0.76 | 0.77 | 0.49 | 0.93 |
| SOFA + C-reactive protein ⑤ | **0.84 (0.76, 0.92)** | 0.75 | 0.82 | 0.81 | 0.55 | 0.92 |
| SOFA + Albumin ⑥ | **0.83 (0.75, 0.91)** | 0.78 | 0.82 | 0.81 | 0.56 | 0.93 |
| SOFA + Lactate ⑦ | 0.81 (0.72, 0.90) | 0.84 | 0.64 | 0.68 | 0.40 | 0.94 |
| SOFA + ①② | **0.89 (0.82, 0.96)** | 0.86 | 0.84 | 0.87 | 0.64 | 0.95 |
| SOFA + ①⑤ | 0.88 (0.81, 0.95) | 0.84 | 0.80 | 0.81 | 0.54 | 0.95 |
| SOFA + ①⑥ | 0.89 (0.83, 0.95) | 0.75 | 0.85 | 0.77 | 0.60 | 0.92 |
| SOFA + ②⑤ | 0.86 (0.78, 0.93) | 0.78 | 0.81 | 0.80 | 0.53 | 0.93 |
| SOFA + ②⑥ | 0.86 (0.78, 0.93) | 0.88 | 0.73 | 0.77 | 0.48 | 0.95 |
| SOFA + ⑤⑥ | **0.86 (0.79, 0.93)** | 0.94 | 0.65 | 0.71 | 0.43 | 0.97 |
| SOFA + ①②⑤ | 0.89 (0.83, 0.96) | 0.88 | 0.85 | 0.86 | 0.62 | 0.96 |
| SOFA + ①②⑥ | 0.90 (0.85, 0.96) | 0.81 | 0.89 | 0.88 | 0.68 | 0.94 |
| SOFA + ①⑤⑥ | 0.90 (0.84, 0.96) | 0.81 | 0.89 | 0.87 | 0.67 | 0.94 |
| SOFA + ②⑤⑥ | 0.87 (0.80, 0.95) | 0.91 | 0.70 | 0.74 | 0.46 | 0.96 |
| SOFA + ①②⑤⑥ | 0.91 (0.85, 0.96) | 0.88 | 0.88 | 0.88 | 0.67 | 0.96 |

*AUC* area under the curve, *PPV* positive predictive value, *NPV* negative predictive value, *SOFA* sequential organ failure assessment.

**Table S3. The added prognostic value of the predictive models as determined by the integrated discrimination improvement index**

| **Predictive model** | **IDI (%)^a^** | **95% CI** | ***P* value** |
| --- | --- | --- | --- |
| SOFA + Prior hospitalization ① + C-reactive protein ② | -3.89 | (10.68, 2.89) | 0.26 |
| SOFA + Prior hospitalization ① + Albumin ③ | -2.91 | (-10.17, 4.36) | 0.43 |
| SOFA + Intra-abdominal source ④ + C-reactive protein ② | -5.72 | (-11.57, 0.13) | 0.55 |
| SOFA + Intra-abdominal source ④ + Albumin ③ | -4.77 | (-11.25, 1.71) | 0.15 |
| SOFA + C-reactive protein ② + Albumin ③ | -7.51 | (-16.38, 1.35) | 0.10 |
| SOFA + ①②④ | 1.83 | (-1.27, 4.93) | 0.24 |
| SOFA + ①③④ | 2.10 | (-1.34, 5.54) | 0.23 |
| SOFA + ①②③④ | 3.84 | (-0.39, 8.08) | 0.23 |

*IDI* integrated discrimination improvement

^a^Compared with the combined risk model of prior hospitalization within six months, intra-abdominal source of infection, and the SOFA score.


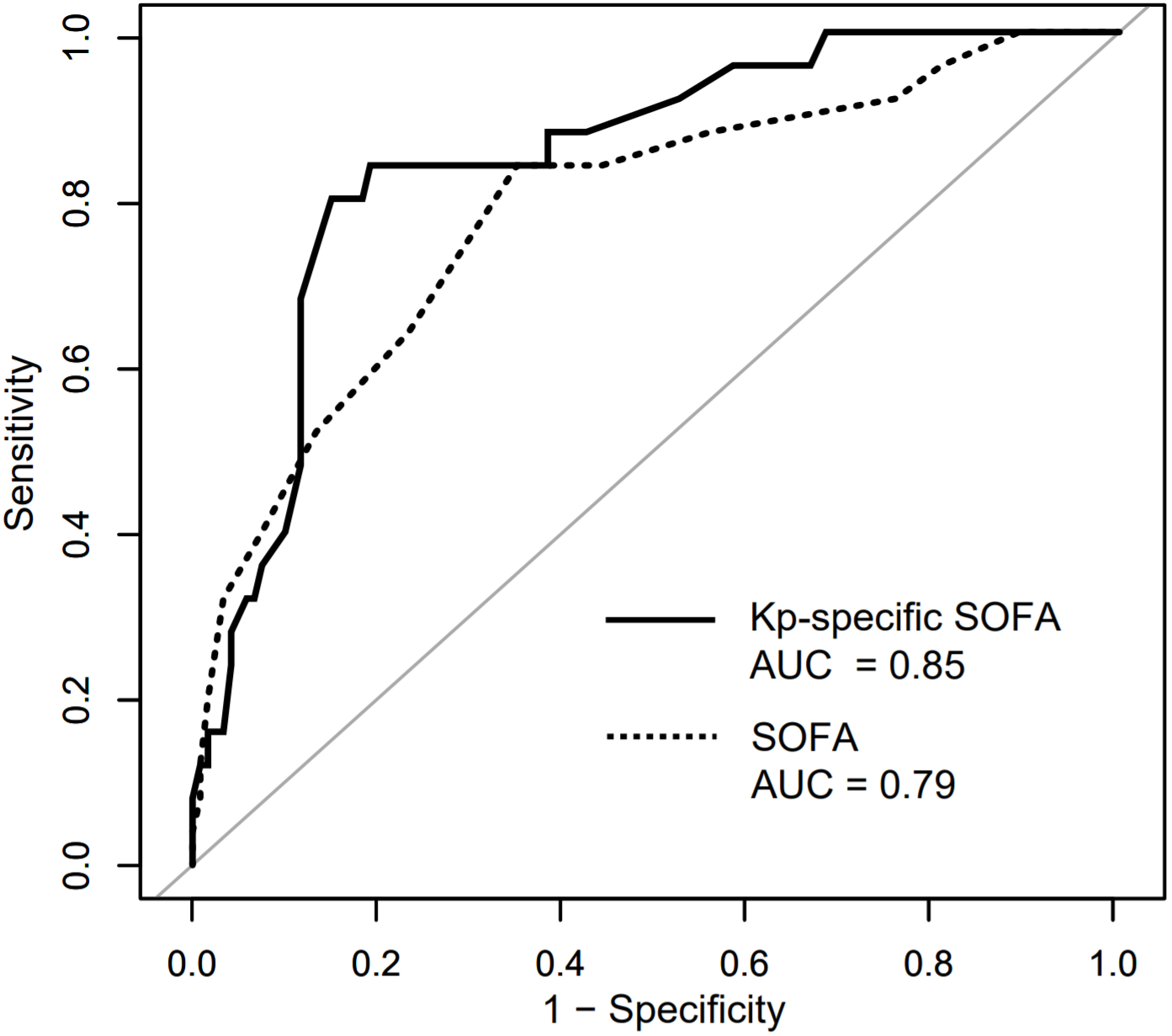


**Fig. S1.** **Receiver operating characteristic curves of the original SOFA score and the Kp-specific SOFA score for predicting 30-day mortality.**


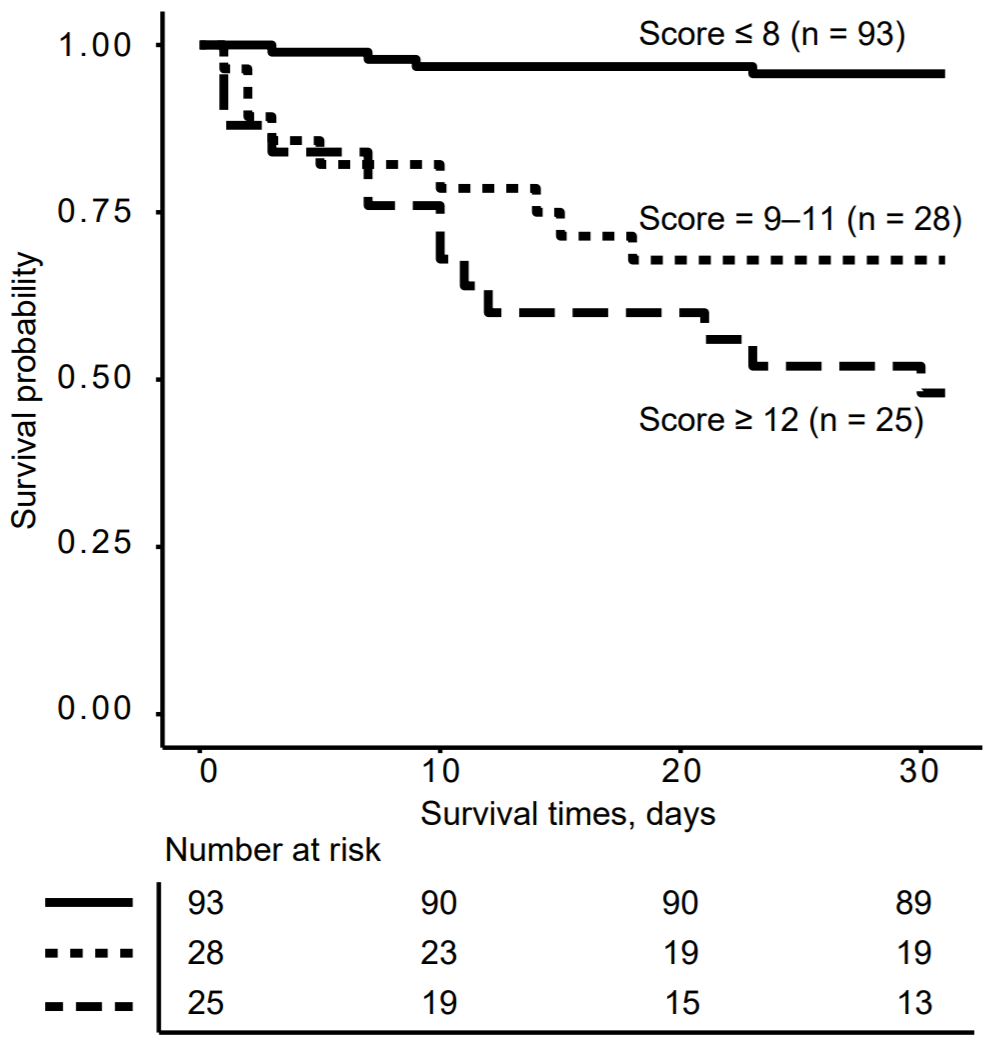


**Fig. S2.** **Thirty-day survival probabilities at three risk categories (score ≤ 8, 9–11 and ≥ 12) after the onset of *Klebsiella pneumoniae* bloodstream infection in children estimated by the Kaplan-Meier analysis (log-rank test, P < 0.01).**
